# Complete genome and transcriptome of *Mycobacterium bovis* 3488, a clinical isolate with a novel deletion at the RD1 locus

**DOI:** 10.1128/mra.00837-24

**Published:** 2025-03-10

**Authors:** Jordy Smith, Maria Manou, Joshua Milgram, Seamus Hoey, Susan Warde, Barbara Kirby, Stephen V. Gordon, Kevin Kenny, Pamela A. Kelly

**Affiliations:** 1 UCD School of Veterinary Medicine, University College Dublin, Dublin, Ireland; 2 Faculty of Agriculture Food & Environment, Koret School of Veterinary Medicine, Hebrew University Jerusalem, Rehovot, Israel; 3 Department of Agriculture, Food and the Marine Laboratories, Celbridge, Kildare, Ireland; University of Notre Dame, Notre Dame, Indiana, USA

**Keywords:** *Mycobacterium bovis*, RD1, tuberculosis

## Abstract

In members of the *Mycobacterium tuberculosis* complex, the RD1 locus encodes a type VII secretion system involved in virulence. Herein, we describe the genome and transcriptomic analysis of *Mycobacterium bovis* 3488, a clinical isolate from a cat, with a 14.2 kb deletion that encompasses the RD1 locus.

## ANNOUNCEMENT

*Mycobacterium bovis* causes tuberculosis (TB) in animals and humans (1). The TB vaccine *Mycobacterium bovis* BCG (BCG) was derived by *in vitro* passage of *M. bovis* (2); deletion of the RD1 locus is a key reason for the attenuation of BCG (3, 4). All clinical *M. bovis* isolates described to date contain RD1 (5–7). Herein, we describe *M. bovis* 3488, isolated from a cat that presented to the UCD Veterinary Hospital (8), that harbors a deletion overlapping the BCG RD1 locus—a feature that complicated its original characterization (8).

*Mycobacterium bovis* 3488 was cultured at 37°C in Middlebrook 7H9-ADC medium with 40 mM sodium pyruvate and 0.05% Tween (9, 10). DNA extraction was performed as previously described (10) with mechanical cell lysis (Roche MagNA Lyser) followed by AMPure XP bead cleanup (Beckman Coulter). For Illumina sequencing, libraries were prepared with Nextera XT Mid output kits and on-bead tagmentation (Illumina) and sequenced on a NextSeq 500 (Illumina) to give 150 bp paired-end reads (125× depth). For Nanopore sequencing, DNA was blunt-end repaired, dA tailed, and adapter ligated using the NEBNext Ultra II DNA kit (New England Biolabs). AMPure XP beads (Beckman Coulter) were used for cleanup and size selection. Libraries were Flongle (ONT) sequenced (R9.4.1) for 24 hours; fastqs were basecalled with Guppy version 6.1.7 using SUP settings (reads 46× depth).

Short read quality was assessed with FastQC version 0.12.0 (11), filtered, and trimmed with BBduk from BBtools version 39 (12). Long reads were filtered and trimmed with Filtlong version 0.2.1 (13) and Porechop version 0.2.4 (14). The depth of sequencing was assessed with BWA (15), Minimap2 (16), and SAMtools (17). Long reads were assembled with Flye version 2.9.2 (18), corrected with Medaka version 1.7.3 (19), and polished with short reads using Polypolish version 0.5.0 (20), POLCA (from MaSuRCA version 4.1.0) (21), and Pilon version 1.24 (22). Contigs were joined and rotated to *dnaA* using Circlator version 1.5.5 (23). The draft sequence was annotated using Prokka version 1.14.5 (24). Assembly quality was assessed using QUAST version 4.0 (25) and with alignments to *M. bovis* AF2122/97 in IGV version 2.16.2 (26). Default parameters were used except where noted (https://github.com/jordysmith/Mbov-omics-annot).

**Fig 1:**
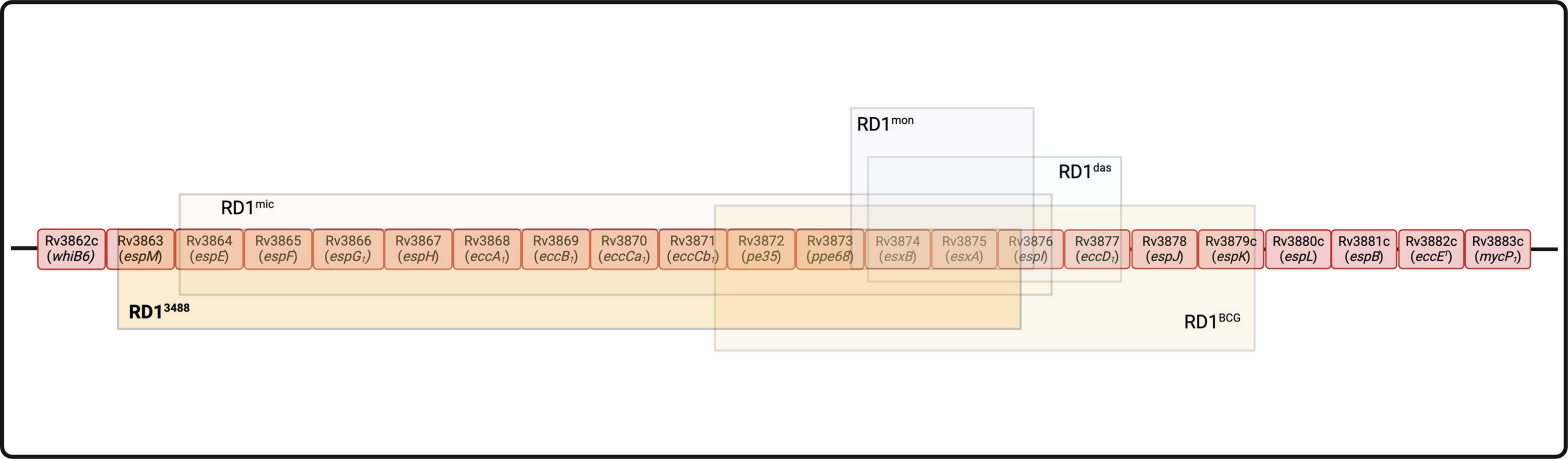
Deletions at RD1 locus across different members of MTBC. RD1^3488^: deletion of *espM′-espI*′ from *M. bovis*^3488^; RD1^mic^: deletion from *Mycobacterium microti*; RD1^das^: deletion from “dassie bacillus”; RD1^BCG^: deletion of RD1 locus from *M. bovis* BCG; and RD1^mon^: deletion from *Mycobacterium mungi* (created with BioRender.com).

The complete genome sequence (Table 1) revealed a deletion of 14,277 bp relative to *M. bovis* AF2122/97 (27), overlapping the BCG RD1 locus, that we designate “RD1^3488^” (Fig. 1).

**TABLE 1:** Genome characteristics

*Mycobacterium bovis* 3488	
Genome size (bp)	4,337,509
No. Illumina reads	3,915,478
No. Nanopore reads	87,050
Nanopore read *N*_50_	3,228
GC%	65.64
Coverage	41×
Contigs	1
Coding sequences	4,031
Pseudogenes	239
rRNA	3
tRNA	52
CRISPR 1	18
CRISPR 2	25

RNA was sequenced commercially (Novogene). Sequence reads were aligned to the *M. bovis* AF2122/97 reference genome (27) with BWA-MEM (15); BAM and GTF were inputted to FeatureCounts (28) to generate gene count data. DESeq2 (29) was used for differential expression analysis. A |log_2_| fold change ≥ 2 and Benjamini–Hochberg adjusted *P* value ≤ 0.05 revealed 44 differentially expressed genes between *M. bovis* 3488 and *M. bovis* AF2122/97. Genes upregulated in *M. bovis* 3488 included the regulators *whiB6* and *espM* and genes flanking RD1^3488^.

Beyond *M. bovis* BCG, deletions at the RD1 locus have been described in *Mycobacterium microti* (30), *Mycobacterium mungi* (31), *Mycobacterium suricattae* (32), and the dassie bacillus (33) (Fig. 1). *Mycobacterium bovis* 3488 is the first *M. bovis* clinical isolate described with a deletion encompassing the RD1 locus.

## Data Availability

The complete assembled genome and annotation for *M. bovis* 3488 were assigned accession number CP139557 following submission to NCBI GenBank with BioProject number PRJNA1019629. Genomic and transcriptomic reads were submitted to SRA with accession numbers SRR26203935 (Nanopore WGS), SRR26203936 (Illumina WGS), and SRR26953547 (RNA-seq).
